# *In Vivo* Antiplasmodial Activity of Different Solvent Extracts of *Myrianthus libericus* Stem Bark and Its Constituents in *Plasmodium berghei*-Infected Mice

**DOI:** 10.1155/2020/8703197

**Published:** 2020-04-21

**Authors:** Michael Kwesi Baah, Abraham Yeboah Mensah, Evelyn Asante-Kwatia, Isaac Kingsley Amponsah, Arnold Donkor Forkuo, Benjamin Kingsley Harley, Silas Adjei

**Affiliations:** ^1^Department of Pharmacognosy, Faculty of Pharmacy and Pharmaceutical Sciences, College of Health Sciences, Kwame Nkrumah University of Science and Technology, Kumasi, Ghana; ^2^Department of Pharmacology, Faculty of Pharmacy and Pharmaceutical Sciences, College of Health Sciences, Kwame Nkrumah University of Science and Technology, Kumasi, Ghana; ^3^Department of Pharmacognosy and Herbal Medicine, School of Pharmacy, University of Health and Allied Sciences, Ho, Ghana

## Abstract

The emergence and resurgence of *P. falciparum* resistance to generations of antimalarial drugs have prompted the search for new, effective, and safe antimalarial agents. This study aimed at investigating the *in vivo* antiplasmodial activity of the 70% hydroethanolic extract and constituents of the stem bark of *Myrianthus libericus* based on its ethnomedicinal use as an antimalarial agent. The antiplasmodial activity was assessed in Swiss albino mice employing the 4-day suppressive and Rane's tests. MLB significantly (*p* < 0.0001) suppressed parasitaemia by 52.26%, 65.40%, and 77.11% at 50, 100, and 200 mg·kg^−1^ doses, respectively, in the 4-day suppressive test. In Rane's test, the highest parasitaemia suppression of 72.50% was recorded at a dose of 200 mg·kg^−1^ of the extract. Fractionation of the bioactive ethyl acetate fraction by solvent-solvent partitioning and column chromatography led to the isolation of friedelan-3-one and stigmasterol being reported for the first time from this species. The compounds demonstrated remarkable antiplasmodial activity by suppressing parasitaemia by 65–72% in the suppressive test and 61–70% in the curative test at doses of 10–30 mg·kg^−1^. Both the extract and the isolated compounds significantly prolonged the survival time of infected mice and averted the cardinal signs associated with *P. berghei*-induced malaria including weight loss, hypothermia, and haemolysis. The results obtained confirm the prospect of *M. libericus* as an important source of new antimalarial compounds and justifies its folkloric use as an antimalarial agent.

## 1. Introduction

Malaria is a life-threatening infectious disease which remains persistent in many regions of the world especially in Africa where a very high prevalence rate of 93% was reported in 2017 [1]. Although recent global statistics show a declining rate in the prevalence and incidence of malaria, the disease is still a problem in Africa as about 395000 deaths were recorded in 2015 [2]. *Plasmodium falciparum* is the parasite responsible for the majority of complicated or fatal malaria cases; however, other species including *Plasmodium malariae*, *Plasmodium vivax, Plasmodium ovale*, and *Plasmodium knowlesi* are known [3].

Over the years, the control and eradication of malaria have been hindered by the emergence and resurgence of *P. falciparum* resistance to several generations of antimalarial drugs [4]. This trend has prompted several research studies which attempt to discover new, effective, and safe antimalarial agents with unique mechanisms of action to combat the resistance pattern of the parasite. Natural products of plant origin have been in the spotlight as potential sources of new effective antimalarial drugs [5, 6] given the notable antimalarial effects of quinine discovered from the bark of *Cinchona rubra* [7] and the artemisinins from *Artemisia annua* which have become essential components of antimalarial therapies [8].

In Ghana, the importance of medicinal plants for the treatment of diseases cannot be overemphasized [9]. Ethnobotanical surveys have identified a number of plants used in traditional medicine for the effective treatment of malaria [10–12]. One of such plants is *Myrianthus libericus* Rendle (Cecropiaceae), popularly known as “*Nyankoma-nini*” (Akan-Asante) in the local Ghanaian language [13].

*M. libericus* is a small tree which grows up to about 10 m on wet and swampy soils in the forest zones of tropical African countries including Guinea, Liberia, and Ghana. It bears simple elliptical green leaves with serrated margins and acuminate apices [14]. The antioxidant properties of the leaves have been reported [15]. Pentacyclic triterpene esters, namely, methyl benthamate, methyl tormentate, methyl arjunolate, methyl euscaphate, methyl 3-isoarjunolate, and methyl 3*β*-O-(4″-O-methyl-E-coumaroyl)-arjunolate, were isolated from the defatted trunk wood [16]. The leaf is reported to be used as an antidote in traditional medicine [17]. In Ghana, the stem bark also finds use as an antimalarial. There is however no report on the antiplasmodial effect of *M. libericus* to the best of our knowledge. The aim of this study was therefore to investigate the antiplasmodial activity of the hydroethanolic stem bark extract, fractions, and bioactive constituents of *M. libericus*.

## 2. Methods

### 2.1. Chemicals

All chemicals and reference drugs were purchased from Sigma-Aldrich Co. Ltd. Irvine, UK. All organic solvents (ethanol (EtOH), petroleum ether (pet-ether), ethyl acetate (EtOAc), chloroform (CH_3_Cl), and methanol (MeOH)) were of analytical grade and obtained from BDH, Laboratory Supplies (Merck Ltd, Lutterworth, UK).

### 2.2. Plant Material Collection

Stem barks of *M. libericus* were harvested from Kwahu Asakraka in the Eastern Region of Ghana in December 2017. The identity of the plant material was confirmed by Dr. George Henry Sam of the Department of Herbal Medicine, Faculty of Pharmacy and Pharmaceutical Sciences, Kwame Nkrumah University of Science and Technology, where a voucher specimen with the code KNUST/HM1/2017/SB014 was deposited at the herbarium.

### 2.3. Preparation of Extract and Fractions

The plant material was carefully washed with water to remove any dirt and soil, cut into smaller pieces, air-dried, and mechanically ground into a coarse powder. The powdered material (3 kg) was Soxhlet extracted with 6 litres of 70% ethanol to obtain an extract which was concentrated using a rotary evaporator (SEO5 rotary evaporator, Australia) under reduced pressure at 40°C and further evaporated to dryness at room temperature to obtain 155 g of semisolid extract referred to as MLB (percentage yield = 5.17%w/w). About 150 g of MLB was dissolved in methanol (150 mL) and successively fractionated by solvent-solvent partitioning to obtain the petroleum ether (MLB-Pet, 11 g), ethyl acetate (MLB-EtOAc, 23 g), and methanol fractions (MLB-MeOH, 61 g) (details of the preparation of fraction are provided in supplementary material). The fractions were stored in air-tight amber coloured glass containers in a refrigerator until needed for use.

### 2.4. Preliminary Phytochemical Screening

The powdered plant material was screened for plant secondary metabolites such as tannins, glycosides, phenolic compounds, alkaloids, sterols, flavonoids, and terpenoids following previously established methods as described by Evans [18].

### 2.5. Animals

Donor albino rats infected with chloroquine-sensitive *Plasmodium berghei* (ANKA strain) and healthy Swiss albino mice weighing between 18 and 26 g were purchased from the Noguchi Memorial Institute for Medical Research (NMIMR), University of Ghana, Accra, Ghana, and transferred to the animal house of the Pharmacology Department, KNUST, Ghana. The animals had access to a standard pellet diet and water *ad libitum*. Laboratory conditions were maintained at 25 ± 1°C, 60–70% relative humidity, and 12 h light-dark cycle. The National Institute of Health Guidelines for Care and Use of laboratory animals (2011) was followed in all experiments. The Pharmacology Department Ethics Committee, KNUST, approved all experimental protocols.

### 2.6. Acute Toxicity Test

The hydroethanolic crude extract of *M. libericus* was investigated for acute toxicity following standard methods [19]. Swiss albino mice of both sexes in groups of five were fasted overnight (water *ad libitum*) and orally administered with a single dose of 50, 500, and 5000 mg·kg^−1^ of extract or normal saline (0.9% 10 mL·kg^−1^). The animals were observed closely for signs of toxicity, behavioural changes, or death at 0, 15, 30, 60, 120, and 180 min, 24 h, and 14 days after extract administration.

### 2.7. In Vivo Antiplasmodial Activity

#### 2.7.1. Parasite Inoculation

Inoculation of plasmodium parasite into healthy albino mice was carried out by a method described by Johnson et al. and the method description partly reproduces their wording [20]. Briefly, donor albino rats infected with chloroquine-sensitive *Plasmodium berghei* with a parasitaemia level of 30–40% were anaesthesized by inhalation of chloroform. Blood samples were then obtained from the donor rats by cardiac puncture using a sterile syringe and transferred into EDTA tubes. Based on the parasitaemia level of donor rats and the red blood cell (RBC) count of healthy mice, the blood samples were diluted with normal saline to obtain a sample with parasitaemia level of 5 × 10^7^ parasitized erythrocytes per mL. Healthy mice for the antiplasmodial experiment were infected by intraperitoneal injection of 0.2 mL of inoculum (containing 1 × 10^7^ parasitized erythrocytes).

#### 2.7.2. The Four-Day Suppressive Test

This test was carried out to investigate the schizontocidal potential of the extract in early infection following the method described by Belay et al. [21]. On the day of the experiment (day 0), infected mice were randomly grouped with 6 mice in each group. Three hours postinfection, the negative control group received 2% Tween 80 solution (p.o.); the treatment groups received: MLB 50, 100, and 200 mg·kg^−1^·day^−1^ (p.o.) or MLB-solvent fractions 200 mg·kg^−1^·day^−1^ (p.o.) or ML1/ML2 10, 20, and 30 mg·kg^−1^·day^−1^ (p.o.), and the positive control group received 2 mg kg^−1^·day^−1^ (i.p.) of artesunate. Treatment was continued for four consecutive days (day 0–day 3) administering samples at the same time each day. On the fifth day (day 4), thin blood smears were collected from the tail of each mouse, fixed with absolute methanol on labelled glass slides, and permanently stained with 10% Giemsa solution to reveal parasitized RBCs. Parasitaemia level was ascertained by counting the number of infected RBCs from 5 randomly selected fields of view under a magnification of ×100 objective lens of a light microscope (Leica ICC50 HD microscope, Hamburg, Germany). The percentage parasitaemia was determined by the following formula:

(1) $$\begin{array}{c} \text{\% parasitaemia}=\frac{\text{number of parasitized RBC}}{\text{total number of RBC counted}}\;\times 100. \end{array}$$

Mean percentage parasitaemia suppression was calculated as follows:

(2) $$\begin{array}{c} \text{\% mean parasitaemia suppression}=\;\left[\frac{\text{A}-\text{B}}{\text{A}}\right]\times 100, \end{array}$$

where A is the mean % parasitaemia in the vehicle-treated group and B is the mean % parasitaemia of the various treatment groups.

#### 2.7.3. Rane's (Curative) Test

The curative ability of the crude extract was determined according to the method description of Belay et al. [21]. Briefly, healthy mice were inoculated by intraperitoneal injection of 0.2 mL inoculum (containing 1 × 10^7^ of parasitized RBCs) on the first day (day 0). After 72 h of infection (day 3), the mice were put into 5 groups with 6 mice in each group. The treatment groups received 50, 100, and 200 mg kg^−1^ p.o. of MLB or 10, 20, and 30 mg·kg^−1^ p.o. ML1/ML2 whereas the negative and positive control groups received 2% Tween 80 solution p.o. (10 mL·kg^−1^) and 2 mg·kg^−1^ p.o. of artesunate, respectively. Treatment continued for 4 consecutive days (day 3–day 6), and parasitaemia levels were monitored by preparing Giemsa-stained thin blood smears from the tails of each mouse on days 3 and 7 for observation under the microscope.

#### 2.7.4. Monitoring of Survival Time

Infected mice were monitored for death by recording the duration (in days) from the time of infection till death for every mouse for a period of 30 days in both suppression and curative assay models [22]. The mean survival time (MST) for the various groups was determined as follows:

(3) $$\begin{array}{c} \text{MST}=\frac{\text{sum of survival time of all mice in a group }\left(\text{days}\right)}{\text{sum of mice in the group}}. \end{array}$$

#### 2.7.5. Determination of Packed Cell Volume (PCV)

The potential of the crude extract to prevent haemolysis that results from increasing parasitaemia levels was investigated by measuring the packed cell volume (PCV). Blood samples were obtained from the tail of each mouse and filled to ^3^/_4_ the volume of heparinized capillary tubes with the dry end sealed. The tubes were centrifuged for 5 minutes at 12,000 rpm in a microhaematocrit centrifuge (Heraeus Biofuge Primo Centrifuge, Hamburg, Germany) with the sealed end outward. The PCV which measures the proportion of RBCs to plasma was determined using a standard microhaematocrit reader [22]. Measurements were done before and after treatment using the following formula:

(4) $$\begin{array}{c} \text{PCV}=\frac{\text{volume of erythrocytes in a given volume of blood}}{\text{total blood volume}}. \end{array}$$

#### 2.7.6. Monitoring of Body Weight

The body weights of the mice were taken before (day 0) and after treatment (day 4) in the four-day suppressive test and on day 3 and day 7 after treatment in Ran's test. The weight of each mouse was taken with the aid of a digital weighing balance (Sartorius, Hamburg, Germany).

#### 2.7.7. Determination of Rectal Temperature

The rectal temperature of each mouse was measured before (day 0) and after treatment (day 4) in the four-day suppressive test and on day 3 after infection and on day 7 after treatment in Rane's test using a digital thermometer.

### 2.8. Data Analysis

Data are presented as mean ± SEM. Comparisons were made between the negative control group and treatment groups as well as the positive control group using one-way analysis of variance (ANOVA) followed by Dunnet's *post hoc* test for multiple comparisons between tests. Mean PCV, rectal temperature, and body weight before and after infection and treatment were compared using two-way analysis of variance followed by Bonferroni's *post hoc* test for multiple comparisons between tests. Results were considered statistically significant at *p* < 0.05. GraphPad Prism 6 for Windows (GraphPad Software, Inc.) was used for all analysis.

### 2.9. Isolation and Identification of Bioactive Constituents

MLB-EtOAc demonstrated the highest suppression of parasitaemia in the 4-day suppressive test and was therefore subjected to purification by chromatography as described by Mireku et al. [23]. Column chromatography was performed using silica gel 60 (70−230 mesh; AppliChem, GmbH, Darmstadt, Germany) by gradient elution with pet-ether, EtOAc, and MeOH, Sephadex LH-20 (25–100 *μ*m). This was followed by thin-layer chromatography (precoated silica gel 60 TLC plates) (GF254 0.25 mm, Alpha Laboratories, UK). Characterization of compounds was performed based on nuclear magnetic resonance (Bruker DRX-500 NMR spectrometer), UV-Vis (PerkinElmer UV/VIS), and infrared (FT-IR, Alpha Brüker, Hamburg) spectroscopic data, and melting point determination (Stuart SMP10 digital melting point apparatus, Bibby Scientific Ltd. Stone, UK) and by comparison with published data. Details of the isolation procedure are presented in the supplementary material.

## 3. Results

### 3.1. Preliminary Phytochemical Screening

The result of preliminary phytochemical screening of the powdered stem bark of *M. libericus* is presented in Table 1.

### 3.2. Acute Toxicity

The hydroalcoholic stem bark extract of *M. libericus* was found not to be toxic at doses up to 5000 mg·kg^−1^. No behavioural or physical changes or death occurred within 24 hours of observation and at 14 days after extract administration. The LD_50_ was therefore above 5000 mg·kg^−1^.

### 3.3. In Vivo Antiplasmodial Activity of Extract and Fractions

#### 3.3.1. Four-Day Suppressive Test

MLB caused a significant decrease in parasitaemia (*p* < 0.0001) at all test doses when compared with the vehicle-treated group. Increasing doses resulted in increased suppression of parasitaemia. The highest suppression of parasitaemia of 77.11% was given by the crude extract, MLB 200 mg·kg^−1^. Among the three solvent fractions tested, MLB-EtOAc gave the highest suppression of parasitaemia of 73.59%. The mean survival times of infected mice treated with MLB were significantly prolonged at all doses of the extract with 200 mg·kg^−1^ dose resulting in the longest survival time of 24 (±0.58) days. The effect of the positive control artesunate was however more significant than the extract and fraction-treated groups. Results are summarized in Table 2.

#### 3.3.2. Effect of MLB on Body Weight, Packed Cell Volume, and Body Temperature in the Suppressive Test

**Figure 1:**
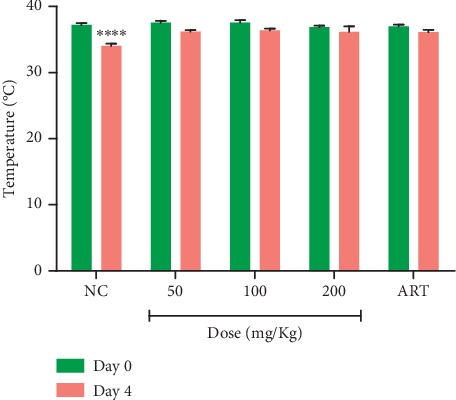
The effect of MLB on body temperature of *P. berghei*-infected mice on day 0 and day 4 in the 4-day suppressive test. Values are presented as mean ± SEM, *n* = 6. NC = vehicle-treated group; ART = artesunate (2 mg/kg). ^*∗∗∗∗*^Values are significantly different at *p* < 0.0001 compared to day 0.

Treatment with MLB averted a significant decrease in PCV and prevented weight loss in infected mice as compared to the vehicle-treated group which showed a significant reduction in body weight and PCV. Similarly, the artesunate-treated group showed no significant decrease in both PCV and body weight after the 4-day treatment (Table 3). At all doses of MLB, artesunate, and vehicle, a reduction of body temperature was recorded. However, the decrease in body temperature was not significant for MLB- and artesunate-treated groups between day 0 and day 4 compared to the vehicle-treated group which showed a significant decrease in rectal temperature on day 4 (Figure 1).

#### 3.3.3. Rane's (Curative) Test

An evaluation of the curative ability of MLB revealed that the extract caused a remarkable reduction in the levels of parasitaemia on day 7 as compared to day 3 at all the doses. The effect was dose-dependent and significant (*p* < 0.0001) compared to the vehicle-treated group. The highest suppression of parasitaemia of 72.50% was given by 200 mg·kg^−1^·day^−1^ of MLB on day 7. Survival time of infected mice was also significantly prolonged at all doses of the extract compared to the vehicle-treated group. However, the curative ability exhibited by artesunate (2 mg·kg^−1^·day^−1^) was much higher (98.22%) than the extract at all doses (Table 4).

#### 3.3.4. Effect of MLB on Body Weight, Packed Cell Volume (PCV), and Body Temperature in the Curative Test

**Figure 2:**
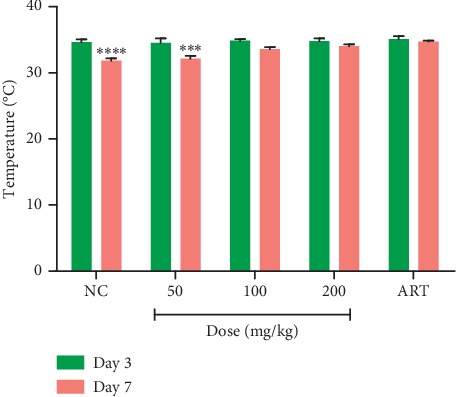
Effect of MLB on body temperature between day 3 and day 7 in the curative test. Values are presented as mean ± SEM, *n* = 6. NC = vehicle-treated group; ART = artesunate (2 mg·kg^−1^). Values are significantly different at ^*∗∗∗*^*p* < 0.001 and ^*∗∗∗∗*^*p* < 0.0001 compared to day 3.

Assessment of the effect of MLB on the change in body weight of *P. berghei*-infected mice in the curative test revealed that, by day 7, MLB at 200 mg·kg^−1^ caused a substantial (*p* < 0.05) increase in body weight when compared to day 3. On the contrary, the vehicle-treated group showed substantial loss in body weight by day 7. Treatment with the standard antimalarial drug artesunate resulted in no weight change. MLB at 100 and 200 mg·kg^−1^ further averted reduction in PCV as well as significant decrease in body temperature on day 7 compared to the vehicle-treated (control) group. The artesunate-treated group showed no significant change in both rectal temperature and PCV (Table 5 and Figure 2).

#### 3.3.5. Isolation and Identification of Bioactive Constituents

**Figure 3:**
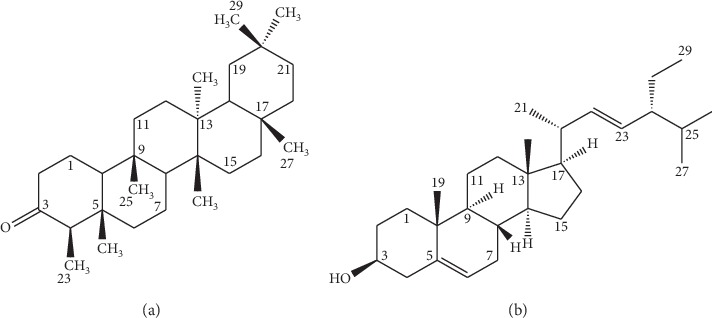
(a) Friedelan-3-one (ML1) and (b) stigmasterol (ML2) isolated from *M. libericus* stem bark.

Bioassay-guided purification of MLB-EtOAc which showed the highest parasitaemia suppression among the three tested fractions resulted in the isolation of two compounds, ML1 and ML2. All spectral and physical data obtained for the compounds matched those reported in the literature for friedelan-3-one (ML1) [24, 25] and stigmasterol (ML2) [26] (Figure 3). The physicochemical constants and NMR spectroscopic data are provided in the supplementary material.

### 3.4. In Vivo Antiplasmodial Activity of ML1 and ML2

#### 3.4.1. Four-Day Suppressive Assay

ML1 and ML2 showed a significant decrease in parasitaemia at all doses when compared to the negative control group though ML1 exhibited a much higher suppression of parasitaemia than ML2. Further, the compounds significantly prolonged the survival time of the mice in a dose-dependent manner. However, no significant effect on the survival time was recorded at 10 mg·kg^−1^·day^−1^ of ML2 (Table 6).

#### 3.4.2. Effect of ML1 and ML2 on Body Weight, Packed Cell Volume, and Body Temperature in the Suppressive Test

**Figure 4:**
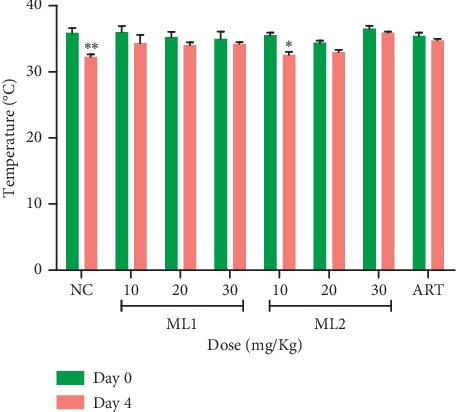
The effect of ML1 and ML2 on body temperature of *P. berghei*-infected mice between day 0 and day 4 in the suppressive test. Values are presented as mean ± SEM, *n* = 6. NC = vehicle-treated group; ART = artesunate (2 mg/kg); values are significantly different at ^*∗*^*p* < 0.05 and ^*∗∗*^*p* < 0.01 compared to day 0.

ML1 and ML2 dose-dependently prevented reduction in body weight, PCV, and body temperature of *P. berghei*-infected mice relative to the negative control group on day 4. Moreover, there was no significant drop in body temperature for mice treated with 20 and 30 mg·kg^−1^ of ML1 and ML2 (Figure 4). ML2 at 10 mg·kg^−1^ however showed a significant (*p* < 0.05) drop in temperature, body weight, and PCV. The artesunate-treated group showed no significant change in body weight, PCV, and temperature (Table 7).

#### 3.4.3. Curative (Rane's) Test

A dose-dependent curative ability was demonstrated by all doses of ML1 and ML2. The compounds significantly (*p* < 0.0001) reduced the level of parasitaemia on day 7 compared to day 3 with ML1 showing better % suppression than ML2. Survival time was significantly prolonged at all doses of the test compounds except for ML2 at 10 mg·kg^−1^·day^−1^ which showed no significant effect on survival time of infected mice. The artesunate-treated group showed 98.22% eradication of the established *P. berghei* infection on day 7 which was higher than the effect of the highest dose of the compounds (Table 8).

#### 3.4.4. Effect of ML1 and ML2 on Body Weight, Packed Cell Volume, and Body Temperature in the Curative Test

**Figure 5:**
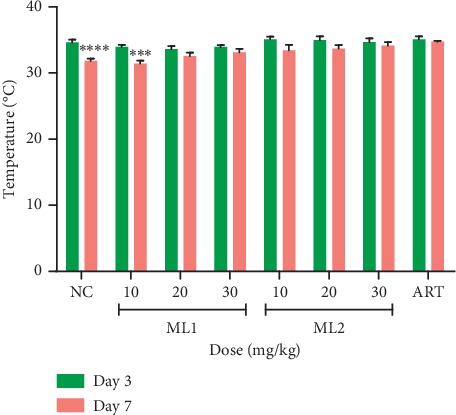
The effect of ML1 and ML2 on body temperature of *P. berghei*-infected mice between day 3 and day 7 in the curative test; values are presented as mean ± SEM, *n* = 6. NC = vehicle-treated group; ART = artesunate (2 mg·kg^−1^); values are significantly different at ^*∗∗∗*^*p* < 0.001 and ^*∗∗∗∗*^*p* < 0.0001 compared to day 3.

Relative to the control group, ML1 and ML2 significantly (*p* < 0.05) averted reduction in body weight, PCV, and body temperature in mice at all doses on day 7 compared to day 3, except for 10 mg·kg^−1^ of ML2 which showed a significant reduction in body weight and body temperature. The artesunate-treated group did not show any significant decrease in body weight, temperature, and PCV (Figure 5). The vehicle-treated group showed a significant decrease in PCV on day 7. Results are presented in Table 9.

## 4. Discussion

In this study, the antiplasmodial activity of the 70% hydroalcoholic extract of the stem bark of *M. libericus* (MLB) and its constituents as well as their effect on the cardinal signs of *P. berghei-*infected mice such as reduction in body weight, hypothermia, and haemolytic anaemia was investigated [27]. *In vivo* antiplasmodial activity models were employed in this study because they take into account the potential effect of prodrugs and the possible contribution of the immune system in fighting the major symptoms of malaria infection [21].

From the acute oral toxicity tests, the LD_50_ of MLB was found to be above 5000 mg·kg^−1^ as no mortality or signs of toxic manifestation such as lacrimation, salivation, convulsion, and immobility were observed at this dose, implying a wide safety margin and partly justifying the safety of the plan in traditional medicine.

The determination of percentage suppression of parasitaemia in the 4-day suppressive test is the standard preliminary model for evaluating antiplasmodial effects against *P. berghei* in mice [28]. MLB demonstrated the ability to reduce the parasitaemia in both early (52–77%) and established infection (50–72%) indicating potential suppressive and curative effects in malaria infection. *In vivo* antiplasmodial activity has been classified as very good, good, and moderate if an extract suppresses parasitaemia by ≥50% at a dose of 100, 250, and 500 mg·kg^−1^·day^−1^, respectively [29]. By this criterion, MLB can be classified as a very good antimalarial candidate. In addition, the extract and fractions significantly prolonged the survival time of mice in both protocols confirming the overall reduction of the pathological effects of parasitaemia in the treated groups [30].

Haemolytic anaemia associated with malaria has been attributed to the phagocytosis of parasitized and nonparasitized RBCs, erythrocytic suppression as well as bone marrow dyserythropoiesis leading to severe disease progression [31]. It is thus important to investigate the potential of an antimalarial agent to prevent anaemia associated with malaria by calculating packed cell volume (PCV). In this study, MLB significantly averted reduction in PCV at all doses in both suppressive and curative assays suggesting the ability of the extract to sustain the production of new RBCs in the bone marrow. Decreased food intake, dysfunctional metabolism, and hypoglycaemia associated with malaria often result in weight loss [32, 33]. Treatment with MLB prevented weight loss in infected mice and rather produced a significant weight gain at 100 and 200 mg·kg^−1^·day^−1^. In a study by Lu et al., artemisinin derivatives were shown to prevent obesity by inducing a process called browning which leads to an improvement of insulin sensitivity, glucose metabolism, thermogenesis, and energy expenditure, leading to weight loss in mice [34]. This may account for a slight loss in weight observed for the artesunate-treated groups. Unlike malaria in humans, *P. berghei*-infected mice experience a decrease in body temperature as a consequence of the haemorrhage in the brain and decreased metabolic rate before their death [35]. Both treated and untreated mice developed some hypothermia in both suppressive and curative tests. However, hypothermia was less pronounced in the treatment group than in the negative control group. The effect of MLB on body temperature, body weight, and haemolytic anaemia in *P. berghei*-infected mice may be attributed to the significant suppression of parasitaemia and the consequent attenuation of the overall pathological effects of the infection in mice [27].

Preliminary phytochemical screening of the stem bark of *M. libericus* revealed the presence of alkaloids, triterpenoids, phytosterols, saponins, tannins, coumarins, and flavonoids. Several studies on the antiplasmodial effect of medicinal plants have been linked to the presence of such plant's secondary metabolites [36–39]. Further phytochemical investigation on the stem bark resulted in the isolation and characterization of a known friedelane-triterpene, friedelan-3-one (ML1), and a phytosterol, stigmasterol (ML2). This is the first report of these compounds from *M. libericus*. The compounds exhibited significant dose-dependent antiplasmodial activity with friedelan-3-one (30 mg·kg^−1^) giving the highest effect in both suppressive (72.28%) and Rane's (70.64%) tests. In previous studies, friedelan-3-one from the root bark of *Harungana madagascariensis* exhibited *in vitro* antiplasmodial activity against W2 strain of *P. falciparum* with an IC_50_ of 7.70 *μ*M [40]. The current result of *in vivo* antiplasmodial activity thus complements the previous *in vitro* report. The antiplasmodial activity of triterpenes has been proposed to be *via* modulation of the cell membrane of nonparasitized erythrocytes, thereby restricting parasites' invasion into healthy RBCs [41, 42]. The antiplasmodial activity demonstrated by isolated compounds confirms the prospect of *M. libericus* as an important source of new antimalarial agents.

## 5. Conclusion

The antiplasmodial activity of the stem bark of *M. libericus* has been demonstrated in this work. The decline in parasitaemia as well as the significant weight recovery and survival rate in the treated mice justifies the folkloric use of the *M. libericus* in the treatment of malaria. This finding also points to the possible presence of other potentially effective antimalarial constituents in the stem bark of *Myrianthus libericus*.

## Figures and Tables

**Table 1:** Phytochemical screening of the stem bark of *M. libericus*.

Secondary metabolite	Result
Tannins	+
Reducing sugars	+
Alkaloids	+
Saponins	+
Triterpenoids	+
Phytosterols	+
Flavonoids	+
Coumarins	+

*Note*. The sign “+” indicates detected.

**Table 2:** Parasitaemia level, % suppression, and survival time of the *P. berghei*-infected mice treated with MLB and solvent fractions in the suppressive test.

Sample	Dose (mg·kg^−1^)	% parasitaemia	% suppression	Mean survival time (days)
NC	10 mL·kg^−1^	67.58 ± 1.31	—	7.00 ± 0.89
MLB	50	32.26 ± 0.57^b4^	52.26	9.83 ± 0.30^b4^
MLB	100	24.18 ± 0.54^b4^	65.40	14.50 ± 0.43^b4^
MLB	200	15.47 ± 1.05^b4^	77.11	24.00 ± 0.58^b4^
MLB-Pet	200	60.05 ± 0.90^b1^	5.62	7.94 ± 0.48
MLB-EtOAc	200	16.80 ± 0.92^b4^	73.59	23.81 ± 0.72^b4^
MLB-MeOH	200	27.93 ± 0.60^b4^	56.11	13.94 ± 0.67^b4^
ART	2	0.75 ± 0.02^b4^	98.89	30.00 ± 0.00^b4^

Values are presented as mean ± SEM, *n* = 6. NC = vehicle-treated group; ART = artesunate. Values are significantly different at ^1^*p* < 0.05 and ^4^*p* < 0.0001, ^b^compared to the vehicle-treated group.

**Table 3:** Effect of MLB on body weight and packed cell volume in the four-day suppressive test.

Dose (mg·kg^−1^)	Body weight (g)			Packed cell volume		
	Day 0	Day 4	Δ*W*	Day 0	Day 4	ΔPCV
NC	23.40 ± 0.81	18.59 ± 0.31^a1^	−4.81	50.90 ± 1.08	45.77 ± 0.66^a3^	−5.13
50	20.95 ± 1.10	20.52 ± 1.21	−0.43	49.95 ± 0.81	47.15 ± 0.23	−2.8
100	21.08 ± 0.67	22.57 ± 0.92	1.49	51.22 ± 1.42	49.82 ± 0.27	−1.4
200	21.32 ± 0.65	22.20 ± 0.74	0.88	50.22 ± 0.97	49.42 ± 0.33	−0.8
ART (2)	20.95 ± 1.64	19.40 ± 2.02	−1.55	49.63 ± 0.89	48.80 ± 0.47	−0.83

Values are presented as mean ± SEM, *n* = 6. NC = vehicle-treated group; ART = artesunate. Values are significantly different at ^1^*p* < 0.05 and ^3^*p* < 0.001, ^a^compared to day 0.

**Table 4:** Parasitaemia level, % suppression, and mean survival time of *P. berghei-*infected mice in the curative test.

Dose (mg·kg^−1^)	% parasitaemia		% suppression	Mean survival time (days)
	Day 3	Day 7		
NC	43.70 ± 1.79	70.80 ± 2.01^a4^	—	6.00 ± 0.93
50	46.35 ± 3.48	35.20 ± 0.61^a4,b4^	50.28	11.33 ± 0.99^b4^
100	47.19 ± 3.10	28.63 ± 1.90^a4,b4^	59.56	13.67 ± 0.49^b4^
200	39.71 ± 1.82	19.47 ± 1.13^a4,b4^	72.50	21.50 ± 0.43^b4^
ART (2)	40.06 ± 2.13	1.26 ± 0.10^a4,b4^	98.22	29.67 ± 0.33^b4^

Values are presented as mean ± SEM, *n* = 6. NC = vehicle-treated group; ART = artesunate. ^4^Values are significantly different at *p* < 0.0001, ^a^compared to day 3, ^b^compared to negative control.

**Table 5:** Effect of MLB on body weight and PCV in the curative test.

Dose (mg·kg^−1^)	Body weight (g)			Packed cell volume		
	Day 3	Day 7	Δ*W*	Day 3	Day 7	ΔPCV
NC	21.18 ± 0.52	18.06 ± 0.41^a1^	−3.12	44.67 ± 0.62	41.95 ± 0.37^a4^	−2.72
50	19.94 ± 1.06	20.32 ± 0.66	0.38	45.43 ± 0.52	43.82 ± 0.62^a1^	−1.61
100	20.68 ± 0.70	21.99 ± 0.84	1.31	48.28 ± 0.21	47.05 ± 0.15	−1.23
200	20.93 ± 0.51	23.79 ± 0.71^a1^	2.86	47.92 ± 0.45	47.28 ± 0.24	−0.64
ART (2)	21.83 ± 0.93	21.22 ± 0.69	−0.61	47.17 ± 0.31	46.95 ± 0.24	−0.22

Values are presented as mean ± SEM, *n* = 6. NC = vehicle-treated group; ART = Artesunate. Values are significantly different at ^1^*p* < 0.05 and ^4^*p* < 0.0001, ^a^compared to day 3.

**Table 6:** Parasitaemia level, % suppression, and mean survival time of *P. berghei*-infected mice treated with ML1 and ML2 in the suppressive test.

Dose (mg·kg^−1^)		% parasitaemia	% suppression	Mean survival time (days)
NC	10 mL·kg^−1^	65.12 ± 2.056	—	9.50 ± 0.76

ML1	10	33.24 ± 0.71^b4^	48.95	11.67 ± 0.67^b1^
	20	25.33 ± 0.65^b4^	61.11	13.33 ± 0.56^b4^
	30	18.05 ± 1.50^b4^	72.28	22.17 ± 0.48^b4^

ML2	10	33.49 ± 0.49^b4^	48.57	10.67 ± 0.49
	20	26.21 ± 0.98^b4^	59.75	12.17 ± 0.60^b2^
	30	22.72 ± 1.21^b4^	65.11	20.50 ± 0.43^b4^
ART	2	0.00 ± 0.00^b4^	100.00	30.00 ± 0.00^b4^

Values are presented as mean ± SEM, *n* = 6. NC = vehicle-treated group; ART = artesunate. Values are significantly different at ^1^*p* < 0.05, ^2^*p* < 0.01, and ^4^*p* < 0.0001, ^b^compared to the vehicle-treated group.

**Table 7:** Effect of ML1 and ML2 on body weight and PCV in the suppressive test.

Dose (mg·kg^−1^)		Body weight (g)			Packed cell volume		
		Day 0	Day 4	Δ*W*	Day 0	Day 4	ΔPCV
NC		21.53 ± 0 .41	17.63 ± 0.69^a2^	−3.9	53.03 ± 1.08	48.86 ± 0.67^a1^	−4.17

ML1	10	21.80 ± 0.569	19.20 ± 0.21	−2.6	51.40 ± 0.81	49.60 ± 0.40	−1.8
	20	22.33 ± 0.731	20.53 ± 0.63	−1.8	54.33 ± 1.42	53.30 ± 0.47	−1.03
	30	21.40 ± 1.22	20.93 ± 0.56	−0.47	52.63 ± 0.97	51.70 ± 0.92	−0.93

ML2	10	23.03 ± 0.83	20.00 ± 0.50^a1^	−3.03	53.37 ± 1.71	49.23 ± 0.58^a1^	−4.14
	20	22.10 ± 1.28	21.37 ± 0.18	−0.73	55.87 ± 0.89	53.37 ± 0.84	−2.5
	30	22.28 ± 0.83	21.87 ± 0.15	−0.41	55.23 ± 1.19	54.17 ± 0.55	−1.06
ART	2	20.93 ± 0.81	19.17 ± 0.30	−1.76	56.17 ± 1.07	55.50 ± 0.47	−0.67

Values are presented as mean ± SEM, *n* = 6. NC = vehicle-treated group; ART = artesunate. Values are significant at ^1^*p* < 0.05 and ^2^*p* < 0.01 , ^a^compared to day 0.

**Table 8:** Parasitaemia level, % suppression, and mean survival time of *P. berghei*-infected mice treated with ML1 and ML2 in the curative test.

Dose (mg·kg^−1^)		% parasitaemia		% suppression	Mean survival time (days)
		Day 3	Day 7		
NC		43.70 ± 1.79	70.80 ± 2.01^a4^	—	6.00 ± 0.93

ML1	10	40.38 ± 1.51	37.39 ± 0.22^b4^	47.19	10.50 ± 0.72^b1^
	20	40.28 ± 1 .72	28.95 ± 1.29^a4,b4^	59.11	11.83 ± 0.30^b3^
	30	41.63 ± 0.76	20.78 ± 1.13^a4,b4^	70.64	20.50 ± 0.56^b4^

ML2	10	41.29 ± 0.54	37.56 ± 0.42	46.95	9.83 ± 0.31
	20	42.16 ± 1.92	30.64 ± 0.67^a4,b4^	56.72	11.50 ± 0.72^b3^
	30	41.97 ± 0.94	27.52 ± 0.89^a4,b4^	61.13	19.67 ± 0.49^b4^
ART	2	40.06 ± 2.13	1.26 ± 0.10^a4,b4^	98.22	29.67 ± 0.33^b4^

Values are presented as mean ± SEM, *n* = 6. NC: vehicle-treated group; ART: artesunate. Values are significantly different at ^1^*p* < 0.05, ^3^*p* < 0.001, and ^4^*p* < 0.0001, ^a^compared to day 3, ^b^compared to negative control.

**Table 9:** Effect of ML1 and ML2 on body weight and PCV in the curative test.

Dose (mg kg^−1^)		Body weight (g)			Packed cell volume		
		Day 3	Day 7	Δ*W*	Day 3	Day 7	ΔPCV
NC		21.18 ± 0.52	18.06 ± 0.41^a2^	−3.12	44.67 ± 0.62	41.95 ± 0.37^a2^	−2.72

ML1	10	20.08 ± 0.43	18.16 ± 0.25	−1.92	45.40 ± 0.53	43.91 ± 0.57	−1.49
	20	20.52 ± 0.63	19.27 ± 0.42	−1.25	47.00 ± 0.48	45.64 ± 0.44	−1.36
	30	22.16 ± 0.72	21.37 ± 0.56	−0.79	47.77 ± 0.60	47.12 ± 0.27	−0.65

ML2	10	21.66 ± 0.71	18.86 ± 0.40^a2^	−2.8	46.56 ± 0.40	44.60 ± 0.62	−1.96
	20	22.32 ± 0.76	20.64 ± 0.46	−1.68	48.45 ± 0.71	46.88 ± 0.61	−1.57
	30	22.93 ± 0.67	21.59 ± 0.30	−1.34	49.08 ± 1.15	48.63 ± 0.25	−0.45
ART	2	21.83 ± 0.93	21.22 ± 0.69	−0.61	47.17 ± 0.31	46.95 ± 0.24	−0.22

Values are presented as mean ± SEM, *n* = 6. NC: vehicle-treated group; ART: artesunate. Values are significantly different at ^2^*p* < 0.01, ^a^compared to day 3.

## Data Availability

The raw data/results from experiments used to arrive at the findings of this study are available from the corresponding author upon request. Previous reports that were used to support this study are cited at relevant places within the text as references.

## Supplementary Materials

Supplementary Materials Supplementary Materials Preparation of solvent fractions Isolation of ML1 and ML2 Physicochemical constants of isolated compounds NMR Spectroscopic data of isolated compounds Table 1: 1H and 13C NMR data for compounds ML1 ‐ ML2
