# Supplementary material for: In Vivo Antiplasmodial Activity of Different Solvent Extracts of Myrianthus libericus Stem Bark and Its Constituents in Plasmodium berghei-Infected Mice
Source: Evid Based Complement Alternat Med. 2020 Apr 21;2020:8703197. doi: 10.1155/2020/8703197 (PMC7191442; doi:10.1155/2020/8703197)
Supplement: Supplementary Materials — Preparation of solvent fractions Isolation of ML1 and ML2 Physicochemical constants of isolated compounds NMR Spectroscopic data of isolated compounds Table 1: 1H and 13C NMR data for compounds ML1 ‐ ML2 [file 8703197.f1.docx]

***In vivo* antiplasmodial activity of different solvent extracts of *Myrianthus libericus* stem bark and its constituents in *Plasmodium berghei*-infected mice**

Michael Kwesi Baah^1^, Abraham Yeboah Mensah^1^, Evelyn Asante-Kwatia^1*^, Isaac Kingsley Amponsah^1^, Arnold Donkor Forkuo^2^, Benjamin Kingsley Harley^3^, Silas Adjei^1^

^1^ Department of Pharmacognosy, Faculty of Pharmacy and Pharmaceutical Sciences, College of Health Sciences, Kwame Nkrumah University of Science and Technology, Kumasi, Ghana.

^2^ Department of Pharmacology, Faculty of Pharmacy and Pharmaceutical Sciences, College of Health Sciences, Kwame Nkrumah University of Science and Technology, Kumasi, Ghana.

^3^ Department of Pharmacognosy and Herbal Medicine, School of Pharmacy, University of Allied Health Sciences, Ho, Ghana.

*Corresponding author: Asante-Kwatia Evelyn

Department of Pharmacognosy, Faculty of Pharmacy and Pharmaceutical Sciences, College of Health Sciences, Kwame Nkrumah University of Science and Technology, Kumasi, Ghana. Email: [eamireku@knust.edu.gh](mailto:eamireku@knust.edu.gh), Telephone: +233246471364

**SUPPLEMENTARY MATERIAL**

**Preparation of solvent fractions**

150 g of the crude ethanol extract (MLB) was re-dissolved in methanol (150 mL;) and successively fractionated into non-polar, semi-polar and polar fractions by solvent-solvent partitioning in a separating funnel beginning with the non-polar solvent, petroleum ether (200 mL x 3), then ethyl acetate (EtOAc, 200 mL x 3). Thus three fractions petroleum ether, EtOAc and Methanol were obtained. The fractions were concentrated on a rotary evaporator to obtain the petroleum ether (MLB-Pet, 11 g), ethyl acetate (MLB-EtOAc, 23 g) and methanol fractions (MLB-MeOH, 61 g).

**Experimental/Methods**

Isolation of ML1 and ML2

The EtOAc fraction of *M. libericus* stem bark (MLB-EtOAc) exhibited the highest *in vivo* antiplasmodial activity amongst the fractions, hence it was selected for further fractionation and characterization of its constituents. Twenty grams (20 g) of the EtOAc fraction was subjected to column chromatography using silica gel 60 (70-230 mesh) as stationary phase and gradiently eluted with petroleum ether, EtOAc and MeOH to obtain 63 fractions (200 mL each). The fractions were bulked based on TLC profiling into 9 fractions (BMF1–BMF9). Fraction BMF1 (Pet. ether: EtOAc, 9:1) yielded white prismatic crystals with yellow pigment which was further dissolved in methanol and subjected to column chromatography and isocratically eluted with CHCl_3_-MeOH (1:1) using Sephadex LH-20 (25-100 μm) as stationary phase to yield compound ML1 (161 mg). Fraction BMF2 (Pet. ether: EtOAc, 8:2) yielded white sheath-like crystals with green pigment which was also further dissolved in methanol and subjected to column chromatography and isocratically eluted with CHCl_3_-MeOH (1:1) using Sephadex LH-20 (25-100 μm) to yield compound ML2 (183 mg).

**Results**

**Physicochemical constants of isolated compounds**

**Compound ML1** (Friedelane-3-one)

White amorphous powder; m.p.: 263-264 °C; UV (in MeOH–H_2_O) λ_max_: 205 nm; IR V_max_ (KBr) cm^−1^: 1711.74; ^1^H NMR (500 MHz in CDCl_3_) and ^13^C (125 MHz in CDCl_3_) NMR data are shown in Table 1; positive EI–MS *m/z* 426.3862 [M]^+^ (calcd. for C_30_H_50_O, 426.3860).

**Compound ML2** (Stigmasterol):

White crystal; m.p.: 168-169 °C; UV (in MeOH-H_2_O) λ_max_: 257 nm; IR V_max_ (KBr) cm^-1^: 3382.77, 2934.32, 1665.24, 838.57; ^1^H (500 MHz in CDCl_3_) and ^13^C (150 MHz in CDCl_3_) NMR data are shown in Table 1; positive EI-MS *m/z* 412.3446 [M]^+^ (calcd. for C_29_H_48_O, 412.3261).

**NMR Spectroscopic data of isolated compounds**

**Table 1: ^1^H and ^13^C NMR data for compounds ML1 - ML2**

|  | **ML1** | | **ML2** | |
| --- | --- | --- | --- | --- |
| **Position** | **δ C** | **δ H *(J in Hz)*** | **δ C** | **δ H *(J in Hz)*** |
| **1** | 22.2 (CH_2_) | 1.97, *ddd*,  1.70, *ddd* | 37.3 (CH_2_) | 1.08, 1.83 |
| **2** | 41.8 (CH_2_) | 2.34, *ddd*,  2.31, *ddd* | 31.7 (CH_2_) | 1.49, 1.80 |
| **3** | 213.1 (C) | - | 71.8 (CH) | 3.49 |
| **4** | 58.2 (CH) | 2.26, *q* | 42.4 (CH_2_) | 2.24, 2.29 |
| **5** | 42.1 (C) | - | 140.8 (C) | - |
| **6** | 41.3 (CH_2_) | 1.55, *d*,  1.31 *d* | 121.7 (CH) | 5.29, *d* (4.7 Hz) |
| **7** | 18.6 (CH_2_) | 1.51, *m*, 1.11, *m* | 32.0 (CH_2_) | 1.53, 1.97 |
| **8** | 53.1 (CH) | 1.42, *dd* | 31.9 (CH) | 1.44 |
| **9** | 37.9 (C) | - | 51.2 (CH) | 0.94 |
| **10** | 59.5 (CH) | 1.56, *m* | 36.5 (C) | - |
| **11** | 35.4 (CH_2_) | 1.46, *m,* 1.38, *m* | 21.1 (CH_2_) | 1.45, 1.49 |
| **12** | 30.5 (CH_2_) | 1.44, *m*, 1.32, *m* | 39.8 (CH_2_) | 1.13, 1.98 |
| **13** | 39.7 (C) | - | 42.4 (C) | - |
| **14** | 38.3 (C) | - | 56.9 (CH) | 1.12 |
| **15** | 32.5 (CH_2_) | 1.48, *m*, 1.24, *m* | 26.2 (CH_2_) | 1.06, 1.55 |
| **16** | 36.0 (CH_2_) | 1.57, *m*, 1.34, *m* | 28.2 (CH_2_) | 1.26, 1.69 |
| **17** | 30.0 (C) | - | 56.8 (CH) | 1.11 |
| **18** | 42.9 (CH) | 1.59, *m* | 12.0 (CH_3_) | 0.70 s |
| **19** | 35.4 (CH_2_) | 1.46, *m*, 1.24, *m* | 19.4 (CH_3_) | 0.96, s |
| **20** | 28.2 (C) | - | 40.4 (CH) | 1.19, *m* |
| **21** | 32.5 (CH_2_) | 1.48, *m*, 1.24, *m* | 19.8 (CH_3_) | 0.94, *d* (6.8 Hz) |
| **22** | 39.3 (CH_2_) | 1.51, *m*, 0.98, *m* | 138.3 (CH) | 5.10, *dd* (8.4, 15.0 Hz) |
| **23** | 6.8 (CH_3_) | 0.89, *s* | 129.3 (CH) | 4.88, *dd* (8.4, 15.0 Hz) |
| **24** | 14.7 (CH_3_) | 0.71*, s* | 51.2 (CH) | 1.53 |
| **25** | 18.3 (CH_3_) | 0.89, *s* | 32.0 (CH) | 1.44 |
| **26** | 20.1 (CH_3_) | 1.02, *s* | 21.1 (CH_3_) | 0.94, *d* (6.1 Hz) |
| **27** | 18.6 (CH_3_) | 1.07, *s* | 21.1 (CH_3_) | 0.94, *d* (6.1 Hz) |
| **28** | 32.1 (CH_3_) | 1.06, *s* | 25.4 (CH_2_) | 1.37 |
| **29** | 30.0 (CH_3_) | 1.21, *s* | 12.2 (CH_3_) | 0.83, *t* (6.0 Hz) |
| **30** | 31.8 (CH) | 0.93, *s* |  |  |
